# Recurrent positional unilateral pulmonary edema following CABG: an uncommon case report

**DOI:** 10.1093/jscr/rjae615

**Published:** 2024-10-05

**Authors:** Aimen Dammak, Mohamed Abdenadher, Faiza Safi, Fatma Mhiri, Nozha Toumi, Sami Kallel, Rania Hammami

**Affiliations:** Cardiovascular Surgery Department, Faculty of Medicine of Sfax, Habib Bourguiba Hospital, AL ain Road, Km 0.5, 3029, Sfax, Tunisia; Alya Clinic, AL ain Road, Km 1.5, 3051, Sfax, Tunisia; Pedagogy Committee of the Faculty of Medicine of Sfax, Pediatric Department, Hedi Chaker Hospital, AL ain Road, Km 0.5, 3000, Sfax, Tunisia; Cardiovascular Surgery Department, Faculty of Medicine of Sfax, Habib Bourguiba Hospital, AL ain Road, Km 0.5, 3029, Sfax, Tunisia; Radiology Department, Faculty of Medicine of Sfax, Habib Bourguiba Hospital, AL ain Road, Km 0.5, 3029, Sfax, Tunisia; Alya Clinic, AL ain Road, Km 1.5, 3051, Sfax, Tunisia; Cardiology Department, Faculty of Medicine of Sfax, Hedi Chaker Hospital, AL ain Road, Km 0.5, 3000, Sfax, Tunisia

**Keywords:** pericardial agenesis, pulmonary edema, chest X-ray, pericardial reconstruction

## Abstract

We reported a case of pericardial agenesis discovered at the age of 60 during coronary artery bypass grafting surgery. However, this anomaly was not treated during the initial surgery. During the post-operatory period, the patient developed recurrent unilateral right pulmonary edema whenever assuming a semi-upright position. We hypothesized that the positional hemodynamic alterations in this patient were related to this rare congenital anomaly. The patient underwent reoperation, 48 hours later, with synthetic pericardial reconstruction and experienced an uneventful recovery during follow-up.

## Introduction

Pericardial agenesis is an exceedingly rare congenital anomaly characterized by the absence of the pericardium, either partially or completely. The reported prevalence of this condition is ~1 in 10 000 autopsies, making it a remarkably uncommon finding in clinical practice [1, 2]. Diagnosing pericardial agenesis can be particularly challenging due to its nonspecific clinical presentation and the need for specific imaging modalities for definitive confirmation. Clinical suspicion often arises when patients present with symptoms related to cardiac displacement, such as positional dyspnea, chest pain, or palpitations, especially in the absence of other cardiac abnormalities [1–3]. We report a case of pericardial agenesis diagnosed during coronary artery bypass grafting (CABG) surgery and highlight the need for, and the approach to treat this cardiac anomaly.

## Case presentation

We reported the case of a 60-year-old male from Libya who presented with debilitating chest pain. He had a history of diabetes mellitus and smoking and had undergone percutaneous coronary intervention of the left anterior descending (LAD) artery with drug-eluting stent, a year ago due to acute coronary syndrome with ST elevation. Diagnostic evaluations revealed necrosis Q waves on electrocardiogram in the anterior leads and elevated troponin levels. The coronarography showed a severe coronary artery in-stent restenosis of 80% in the LAD, diffuse and long severe stenosis in the circumflex artery, and 70–80% stenosis in the right coronary artery (RCA). Transthoracic echocardiography (TTE) showed a reduced left ventricular ejection fraction (LVEF) of 30% with anterior wall hypokinesia, and arterial pulmonary artery pressure of 35 mmHg.

**Figure 1:**
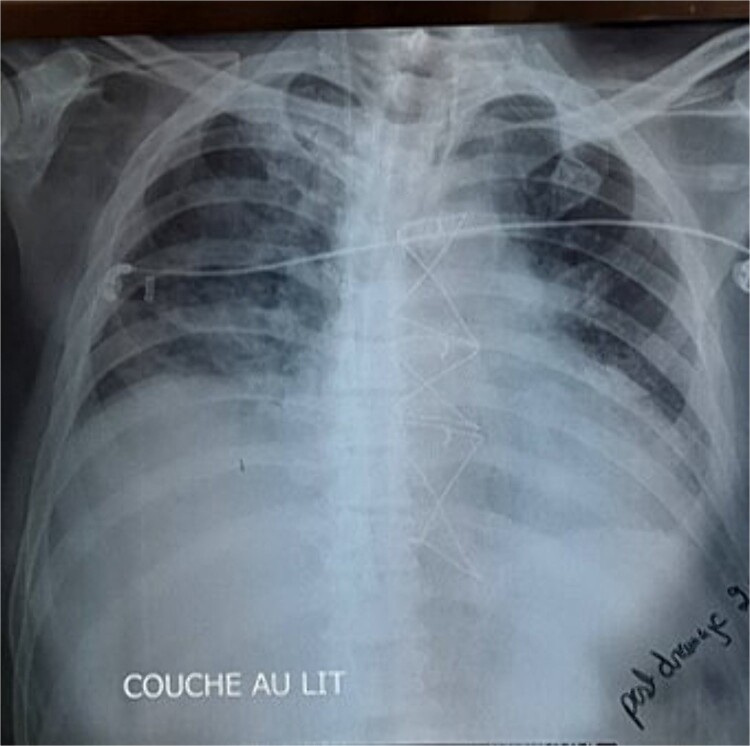
The chest X-ray showed right pulmonary edema, with total deviation of the heart to the left, as well as an elongation and straightening of the left heart border.

Given these findings, CABG was deemed necessary, with plans to place the left internal mammary artery (LIMA) to the LAD, right internal mammary artery (RIMA) to the RCA, and a saphenous vein graft to the first marginal. Intraoperatively, while dissecting the LIMA graft, total pericardial agenesis was unexpectedly discovered, manifesting as a collapsed parietal pleura and total leftward heart deviation. We performed uneventfully the CABG and we decided to not treat the pericardial agenesia. Postoperatively, the patient underwent extubating on the same day but developed non-sustained tachycardia on the electrocardiogram and recurrent unilateral pulmonary edema, particularly notable when he assumed a semi-upright position. The chest X-ray showed right pulmonary edema (Fig. 1), with total deviation of the heart to the left, as well as an elongation and straightening of the left heart border. The right cardiac border was also absent. The TTE showed an LVEF of 30%, high pulmonary arterial pressure = 50 mmHg. We hypothesized that this positional and unilateral pulmonary edema was related to the torsion of the heart and the elongated right pulmonary vein, caused by the deviation of the heart to the left due to the lack of pericardium.

We decided to perform another surgery. The patient underwent a repeat surgery 48 hours after the initial procedure. We constructed a synthetic pericardium using an INTRAMESHⓇ patch (300 × 300 mm). The patch was meticulously sutured using 1/0 non-absorbable polyester suture in a widely spaced interrupted fashion from the posterior mediastinum, starting at the level of the left pulmonary artery, extending posterior to the left atrial appendage, along the left hilum and inferior pulmonary ligament, and down to the diaphragm. The patch was then brought anteriorly to the chest wall and secured along its entire anterior surface, leaving the superior end free (Video 1). The rectangular patch was shaped to create a concavity, allowing space for the base of the heart, including the left atrium and pulmonary veins. The patch was threaded under the apex of the heart, with the two straps sewn to the right and left sternal crests. The remainder of the patch was then sutured to the endothoracic fascia to suspend the heart’s apex forward. The phrenic nerve was preserved; it was adjacent to the mediastinal pleura and outside the patch. This comprehensive reconstruction aimed to stabilize the heart’s position.

Postoperatively, the patient reported no chest pain, and the TTE showed an improved LVEF of 35%. The CT scan performed 1 month later showed that the synthetic pericardium was in place around the heart, ensuring structural integrity and stability.

## Discussion

We reported a new case of pericardial agenesis, unexpectedly discovered at the age of 60 years, during CABG surgery. This case highlighted the difficulty in confirming this congenital anomaly due to its nonspecific symptoms and rarity [1–4]. Pericardial agenesis can be asymptomatic or present with positional symptoms and should be considered a differential diagnosis for atypical, angina-like pain with non-obstructive coronary arteries and evidence of cardiac mobility. On examination, a significantly misplaced apical impulse and systolic ejection murmurs likely from excessive heart motion can be noted. The partial form is more frequently symptomatic [5]. The absence of the left side of the pericardium is the most common anomaly with a prevalence of 70% while the complete absence of pericardium or the absence of the right side of the pericardium are uncommon [5–7]. In our patient, previous symptoms were attributed to coronary disease, and the diagnosis was missed despite TTE and chest X-ray before surgery. Indeed, the left pleura was supporting the heart before the surgery, which is why the patient was asymptomatic. After the CABG, the heart was detached from the pleura and underwent a leftward torsion and that’s why he developed symptoms especially in a supine position.

Moreover, our patient had a RIMA to RCA bypass graft. Given the location of the graft, a left heart deviation could cause impingement of the graft. RCA occlusion can cause right ventricular ischemia and lead to right heart failure, which can cause the patient’s pulmonary edema [7].

A variety of imaging modalities can be helpful for the evaluation and diagnosis of congenital absence of the pericardium. Typical chest X-ray findings, particularly for a left complete pericardial defect, include loss of the right heart border, a leftward shift and straightening of the left cardiac border (Snoopy sign), and lung tissue between the left hemidiaphragm and the base of the heart as well as between the aortic knob and main pulmonary artery [8].

There is no consensus on managing such patients. Generally, treatment is not necessary for asymptomatic patients with a complete unilateral or bilateral pericardial defect, as there is a low risk of complications and an excellent prognosis. Enlarging the defect, patch sealing the defect, pericardiectomy, and pericardioplasty are among the treatment options that often demand a multidisciplinary team effort [9]. Although this is a rare pathology, the surgeon must be aware that after cardiac surgery, the patient could develop symptoms if a pericardial reconstruction has not been done. The heart will be more deviated to the left, causing torsion and traction on the right vessels, especially the right pulmonary veins. This is why our patient developed recurrent positional unilateral edema, which was favored by the semi-upright position. The pericardium plays a crucial role in maintaining cardiac structure and function, providing mechanical protection and facilitating normal cardiac motion. The absence of this protective membrane can lead to various clinical manifestations, often posing diagnostic challenges.

## Conclusion

This case underscores the challenges in diagnosing pericardial agenesis, which can be identified later in adult patients, and the need to correct this anomaly when discovered during cardiac surgery.

## Supplementary Material

video_agnesie_avi_rjae615
